# Supplementary material for: Impact of alexithymia, speech problems and parental emotion recognition on internalizing and externalizing problems in preschoolers
Source: PLoS One. 2024 Sep 10;19(9):e0310244. doi: 10.1371/journal.pone.0310244 (PMC11386473; doi:10.1371/journal.pone.0310244)
Supplement: Attachment — Submitted filename: Response to Reviewers.docx [file pone.0310244.s001.docx]

**Reviewer #1:**

The article provides a compelling examination of alexithymia in preschool-aged children, an area that has been scarcely explored in the literature despite its significant implications for mental health. The study addresses a notable gap by investigating the relationship between child and parental alexithymia and its impact on early childhood psychopathology.

The research is well-written, presenting a clear methodology involving data from 174 parents of children aged 3 to 6. The use of validated questionnaires to assess alexithymia and psychopathology in both children and parents is a strong point, as it allows for a robust analysis of the relationships between these variables. The findings highlight the predictive power of parental alexithymia and emotion recognition abilities on child alexithymia, and how these factors relate to internalizing and externalizing symptomatology in children.

Notably, the study underscores the importance of alexithymia as a potential risk factor in early childhood, providing a foundation for future research aimed at understanding its causes, prevention, and intervention strategies for associated psychopathology. This perspective opens new avenues for exploring early intervention techniques and enhancing parental support programs to mitigate the development of mental health issues in children.

The article's limitations are punctually addressed, further strengthening its validity. The authors' thorough discussion of these limitations ensures that the conclusions drawn are well-supported and that the study provides a comprehensive overview of the topic.

Overall, this study is a valuable contribution to the field, offering fresh insights and setting the stage for future research on alexithymia and childhood psychopathology.

Dear Reviewer 1,

Thank you for your thoughtful and encouraging review of our manuscript. We are delighted that you found our study to be a compelling examination of alexithymia in preschool-aged children and its significant implications for mental health. Your comments on the importance of alexithymia as a potential risk factor in early childhood and the need for future research on its causes, prevention, and intervention strategies are particularly encouraging.

Sincerely,

The authors

**Reviewer #2:**

the article titled Impact of alexithymia, speech problems and parental emotion recognition on
internalizing and externalizing problems in preschoolers is an innovative topic and difficult to conduct for preschoolers too. It will be the foundational stone for further related studies.

Dear Reviewer 2,

Thank you for your positive feedback on our manuscript titled “Impact of Alexithymia, Speech Problems, and Parental Emotion Recognition on Internalizing and Externalizing Problems in Preschoolers." We are pleased that you found the topic innovative and recognized the challenges of conducting such research with preschoolers.

Your acknowledgment of our study as a foundational stone for future related studies is greatly appreciated. We hope our work will inspire and facilitate further research in this important area.

Sincerely,

The authors

**Reviewer #3:** The article presented by the authors aims to fill in the gaps on the topic of alexithymia, where research is scarce. The subject is complex, but the author has produced a very detailed and meticulous article. Previous articles have mainly looked at the adult or pre-school age, leaving out early childhood, which turns out to be extremely important and little studied, given the difficulty of the patient. In addition to congratulating the authors for having written an article on a very complex subject, providing data and considerations that are very important for the entire scientific community, I would like to make the following suggestions to improve an article that is already of a very high standard.
The objectives set by the authors have been respected and developed in an appropriate manner, providing an important starting point for future studies. First of all, the entire article should be reviewed in English, as there are redundant terms that can be improved by synonyms, if not grammatical errors that can be easily corrected.

Dear Reviewer 3,

Thank you very much for the thorough and encouraging review of our manuscript titled “Impact of Alexithymia, Speech Problems, and Parental Emotion Recognition on Internalizing and Externalizing Problems in Preschoolers”, submitted for publication in PLOS ONE. We have thoroughly reviewed the manuscript to address grammatical errors and enhance readability by refining the use of synonyms where appropriate. We are very grateful for your helpful suggestions. Please find a detailed point-by-point response below. The respective changes in the manuscript are highlighted with track-changes to facilitate the follow-up. Page and line numbers refer to the track-changes document with changes visible.

Sincerely,

The authors

Introduction:

38-44: I would pay more attention to this paragraph, as it is of particular importance for the purpose of the article, I would improve its drafting, including any studies, regarding the identification of "pathology" when caregivers struggle to identify and interpret the child's emotions, identifying possible and viable solutions to remedy this problem.

Thank you very much for this important suggestion. Parent-reports on children’s psychopathology, especially in preschool children, are reported to be stable and reliable with few effects of confounding variables. Unfortunately, to our knowledge, there has only been a single study investigating the impact of parental alexithymia on parental report of children’s emotional skills. Thus, we have elaborated on the conducted study and emphasized its implications. As suggested, we have pointed out that assessing these variables is a possible solution to control for their impact (page 5, lines 94 – 105).

Study design:

The study design described in the introduction was explained in a clear and simple manner.

Thank you very much.

Materials and methods:

The flow of data collection, with the criteria used for the study, has been described in an appropriate and thorough manner, there are no corrections to be made as they are meticulous. The statistical data produced are meaningful, clear and easy to understand.

Thank you for your appreciation.

I would suggest an extension of the study court, for future implementation of the study, as it is often and scientifically important.

Thank you very much for pointing this out. The purpose of the present study was to gain a first glance at alexithymia in preschool age in a sample with sufficient power for the conducted analyses. Nevertheless, we fully agree that an extension of the sample size would benefit our conclusions and are planning a future follow up study with more objective measures of child alexithymia and a larger sample size.

The material presented for data collection, was well documented, described it impeccably, there are no corrections to be made.

Thank you very much.

Statistical analysis, well documented, highlighting the strengths and limitations of the study.

We are grateful for the positive feedback.

Conclusions:

The article is very long, but the importance of the topic justifies the length. All data, results and discussions are well documented. The article is of high scientific importance, a necessary result for the whole scientific community, with the aim of increasing knowledge in a topic that is still little explored. All data and methods used are user-friendly. The authors have presented an article that adheres to appropriate reporting guidelines and community standards for data availability. Applicable standards for study ethics and research integrity are met. The manuscript is well written and I believe it can be accepted for publication. Implementation with a series of case study articles is necessary for the future development of the topic of scientific interest, as I believe it will benefit the entire scientific community.

Thank you for your positive evaluation of our article. We appreciate your recognition of the importance and thoroughness of our work, and we are pleased that you find the manuscript suitable for publication. We will certainly consider your suggestion for future studies to further explore this important topic.

**Reviewer #4:**

Dear Reviewer 4,

Thank you very much for the thorough and helpful review of our manuscript titled “Impact of Alexithymia, Speech Problems, and Parental Emotion Recognition on Internalizing and Externalizing Problems in Preschoolers”, submitted for publication in PLOS ONE. We have revised the manuscript to address your helpful suggestions. Please find a detailed point-by-point response below. The respective changes in the manuscript are highlighted with track-changes to facilitate the follow-up. Page and line numbers refer to the track-changes document with changes visible.

Sincerely,

The authors

1. In the abstract of the original article, the details of the materials and methods should be presented separately from the results. This ensures clarity and facilitates understanding of both the procedures employed and the findings obtained.

Thank you very much for this suggestion. We have gladly adjusted the abstract to include separate sections for methods and results (page 2, lines 26 – 32)

2. In Table 2 and 3: Maintain consistency in the notation and format of numerical data. The use of zero as an integer and then omitting it elsewhere could cause confusion. Uniformity in the presentation of results.

Thank you for this suggestion. In our tables we have followed the APA Style requirements which recommend removing leading zeros for values that cannot become larger than 0 (e.g., p-values). We have now adjusted the notation across tables, ensuring leading zeros are included throughout.

3. In the final paragraph of the discussion, spaced 9-10, it is noted that authors are cited in a format different from the Vancouver style. Unify the citation style with the rest of the content.

Thank you very much for spotting this. We have adjusted the citation format to ensure uniformity.

4. The conclusion focuses more on the relevance of the study and its implications for future research, rather than detailing specific findings that address the purpose of the study.

This is an important remark and we have adjusted the conclusion to include detailed findings and implications for future research as a follow-up (page 29, lines 536 – 550).

**Reviewer #5:** Interesting study from the point of view of the mental health of parents and children, however there are some observations that I detail below:

Dear Reviewer 5,

Thank you very much for the thorough and helpful review of our manuscript titled “Impact of Alexithymia, Speech Problems, and Parental Emotion Recognition on Internalizing and Externalizing Problems in Preschoolers”, submitted for publication in PLOS ONE. We have revised the manuscript to address your helpful suggestions. Please find a detailed point-by-point response below. The respective changes in the manuscript are highlighted with track-changes to facilitate the follow-up. Page and line numbers refer to the track-changes document with changes visible.

Sincerely,

The authors

Summary: It is missing to place the study design and briefly the selection criteria. The wording of the statistics used is not understood.

Thank you very much for pointing this out. We have gladly incorporated information on the study design (online survey), the selection criteria (attending regular preschool) and included information on the statistical method used (multivariable linear regressions). Please see page 2, lines 26 – 34.

Introduction: Improve the wording by being more precise with what has been found in the review of the scientific evidence, improve the wording of the justification, and should conclude with the objective of the study.

We are grateful for the provided suggestions and have overworked the introduction to improve wording and formulations. We added additional sentences to point out the justification of the study (i.e., extensive research suggestion alexithymia as a risk factor but no research on alexithymia in preschool children; page 4, lines 77 – 84). The introduction now ends with explicit mention of the objective of the study and our hypotheses.

Material and method: Adequately describe the selection criteria, adequately detail the procedural phase, be more precise with the wording of the statistical analysis.

Thank you for this recommendation. We have extended our description of the selection criteria which were being the parent of children aged 3 to 5, the child attending a regular preschool, absence of intellectual disability, and adequate comprehension of the German language (page 7, lines 156 – 159). Additionally, we have elaborated on the procedural phase and recruitment (page 8, lines 171 – 175) and the statistical analysis (page 11, lines 242 – 256).

Results: Improve the wording of the interpretation of the results.

We have gladly implemented this recommendation and overworked the results, including more details and specifics in the descriptions (pages 12-16, lines 258 – 336).

Conclusion: Be more precise and objective in the wording.

Thank you for pointing this out. We have overworked the conclusion to involve more information on the objective results of the study with a brief outlook at possible implications and future research (page 29, lines 536 – 550).

**Reviewer #6:** The study presents an important and under-researched area in understanding alexithymia in early childhood and its implications for psychopathology, providing valuable insights into the potential intergenerational transmission of emotional processing difficulties. While the topic is relevant and the research question is well-framed, there are several methodological and interpretative concerns that need to be addressed to strengthen the manuscript.

Dear Reviewer 6,

Thank you very much for the thorough and encouraging review of our manuscript titled “Impact of Alexithymia, Speech Problems, and Parental Emotion Recognition on Internalizing and Externalizing Problems in Preschoolers”, submitted for publication in PLOS ONE. We have revised the manuscript to address your helpful suggestions. Please find a detailed point-by-point response below. The respective changes in the manuscript are highlighted with track-changes to facilitate the follow-up. Page and line numbers refer to the track-changes document with changes visible.

Sincerely,

The authors

Major Comments:
Sample Size and Generalizability:
The sample size of 174 parents, including a subgroup of 27 children in an intervention program, raises concerns about the generalizability of the findings. More information on how these participants were selected and how representative they are of the broader population is necessary. Consider discussing the limitations of the sample size more explicitly and how it might impact the study's conclusions.

Thank you very much for pointing out this concern. Regarding the sample size itself we have conducted a power-analysis and determined that our sample size supersedes the 118 participants necessary to be able to draw meaningful statistical conclusions. However, our sample constitutes children in regular preschools in northeastern Bavaria, Germany, and generalizability to samples that do not attend preschool or with different cultural backgrounds may be difficult. We have added information on the selection of participants in the methods section and pointed out that all children attending regular preschools in northeastern Bavaria, Germany, were able to participate (page 8, lines 171 – 175) and included a discussion of the generalizability in the limitations (page 27, line 499 – 506). Additionally, we have added higher parental education as a point that may reduce generalizability in the limitations (page 28, lines 507 – 510).

Measurement Tools:
The reliance on parent-reported measures (Perth Alexithymia Questionnaire – Parent Report and Strengths and Difficulties Questionnaire) introduces a potential bias. Parents’ perceptions may not accurately reflect the child’s true emotional state or behavior.
It would strengthen the study to include direct assessments of the children, possibly through observational methods or clinician-administered measures.

Thank you for your insightful comment. We recognize the concern regarding our reliance on parent-reported measures in this study. Several factors influenced our decision not to include direct assessments of the children:

Firstly, our participants were very young, aged between 3 to 6 years, which posed challenges for conducting direct assessments using observational methods or clinician-administered measures due to their limited cognitive and linguistic abilities. Moreover, in early childhood, parents serve as reliable informants about their children's emotional and behavioural characteristics. Their close involvement in daily interactions provides valuable insights into their emotional development, making parent-reported measures a practical and informative approach for our initial exploration of alexithymia in this age group (pages 4-5, lines 85– 88).

Secondly, objective measures specifically designed for assessing alexithymia in preschool-aged children are currently limited, necessitating the use of validated parent-report questionnaires as the most feasible option (page 9, likes 193 – 194).

Our study aimed to provide an initial insight into alexithymia among preschool-aged children, and thus we considered parent-report measures as a valuable starting point.

We acknowledge these limitations and recognize the importance of integrating direct assessments in future research to complement parental reports and offer a more comprehensive understanding of children's emotional and behavioural functioning. Your feedback is greatly appreciated, and we will take these considerations into account for future research endeavours.

Intervention Program Group:
The inclusion of 27 children in an interdisciplinary intervention program could introduce confounding variables. The differences between this subgroup and the rest of the sample should be clarified.
An analysis comparing outcomes between children in the intervention program and those not in the program would be beneficial.

Thank you for your valuable feedback. The inclusion of children in an interdisciplinary intervention program (IIP) was intentional, based on the hypothesis that these children might exhibit higher levels of psychopathology, thereby adding variance to our sample. We have consistently controlled for IIP participation in our analyses to account for potential confounding effects.

To address your concern, we repeated our analyses excluding the 27 children from the IIP subgroup and confirmed that the results followed the same pattern (page 16, lines 335 – 337). Additionally, we have provided detailed comparative information on the two groups in Table 1 (pages 12 – 13) and significant differences between the groups are reported at the beginning of the results section on page 12, lines 258 – 271. This comparison helps clarify the impact of IIP participation on our findings.

Causality and Directionality:
The cross-sectional nature of the study limits the ability to draw causal conclusions. While the study suggests correlations between parental alexithymia, child alexithymia, and psychopathology, it cannot establish causality.
Consider discussing longitudinal designs or other methods that could be used in future research to better understand the causal relationships.

Thank you very much for emphasizing this. While it is assumed that primary alexithymia is present early on and before the onset of mental health problems, the present study cannot draw any conclusions about causality. We have included this in the limitations and discussed the need for longitudinal designs that observe alexithymia and psychopathology in preschool children over time (pages 28-29, lines 528 – 534).

Minor Comments:
Literature Review:
The introduction could benefit from a more thorough review of existing literature on alexithymia in early childhood and its links to psychopathology to provide a stronger foundation for the study.

Thank you for pointing this out. Unfortunately, the existing literature on alexithymia on early childhood is very limited and constitutes the motivation for conducting the study we are reporting on. We have elaborated on this research gap. Additionally, we have conducted an additional literature search and identified two studies that focus on parental alexithymia (page 4, lines 77 – 81).

Clarification of Terms:
Terms such as "internalizing symptomatology" and "externalizing symptomatology" should be clearly defined for readers who may not be familiar with these concepts.

We have gladly incorporated this recommendation and included clear definitions for the two terms on page 10, lines 223 – 225.

Formatting and Writing:
Ensure consistency in the use of terms and abbreviations throughout the manuscript.
Minor grammatical and typographical errors should be corrected to improve readability.

Thank you for your feedback. We have edited the manuscript to ensure consistency in the use of terms and abbreviations throughout the manuscript. Additionally, we carefully reviewed the text to correct any minor grammatical and typographical errors to enhance readability.

Conclusion:
While the study addresses a significant gap in the literature and provides preliminary evidence on the role of alexithymia in early childhood, the methodological and interpretative concerns need to be addressed to ensure the findings are robust and generalizable. Further research with larger, more representative samples and longitudinal designs is recommended to build on these initial findings.

Thank you for your thoughtful feedback and constructive insights. We have carefully considered and addressed the methodological and interpretative concerns raised to ensure the robustness and generalizability of our findings. Your suggestion for future research with larger, more representative samples and longitudinal designs aligns with our goals, and we aim to build upon these initial findings in subsequent investigations.
